# Supplementary material for: Primary care physician perspectives on screening for axial spondyloarthritis: A qualitative study
Source: PLoS One. 2021 May 24;16(5):e0252018. doi: 10.1371/journal.pone.0252018 (PMC8143395; doi:10.1371/journal.pone.0252018)
Supplement: S1 Table — (DOCX) [file pone.0252018.s002.docx]

**S1 Table. Physician interview questions.**

| **Objective** | **Questions** |
| --- | --- |
| **Experiences with patients with back pain** | What is your overall experience with patients presenting with back pain? |
| **Approach to patients with back pain** | How do you work up patients with back pain?Specifically, how do you work up patients with inflammatory back pain?  - What guidelines or tools do you use?  - How do you use ancillary clinical personnel to assist? |
| **Differential** | What conditions do you consider in your differential for patients with back pain?  Probes (if needed): enthesitis, fibromyalgia, cancer, labs, uveitis sleep, depression, x-rays, history  - What about younger patients (18-30 years) vs older patients (30-45 years). |
| **Axial spondyloarthritis in differential** | If a patient presents with back pain, what would make you consider inflammatory back pain or axial spondyloarthritis in the differential?  - why would it be on your radar diagnostically? Or not?  - would your thinking or approach be different for men vs women? If yes, why?  - would your thinking or approach be different for younger (18-30 years) vs. older (30-45 years) patients?  Do you order HLA-B27 testing, x-rays, or advanced imaging when you see patients for back pain? Why or why not? |
| **Referral** | When do you refer patients with back pain?  - to whom? - why or why not? |
| **Delay in diagnosis** | What if I told you that on average delay in diagnosis for someone with any form of axial spondyloarthritis ranges from 7 to 10 years?  - do you think this is a problem?  - why do you think the delay is so long?  - what do you think should be done to improve dx and reduce the delay? |
| **Implementing a screening tool** | Take a look at this sample screener – what do you think about it?  - would you change any of these questions?  - how likely are you to screen using these questions?  - under what circumstances would you use the screener?  - what are the barriers to screening? how can they be overcome?  - how would you address time constraints?  - overall, what would be the best way for primary care clinicians to screen for inflammatory back pain or axial spondyloarthritis? |
| **How does primary care view axial spondyloarthritis?** | Among primary care practice physicians I general, are they aware of axial spondyloarthritis? Do they know about it? Would they screen for it? In your estimation, how aware are other primary care clinicians? What would it take to raise awareness? What are the barriers? How do you see your role in screening? |
| **Advice** | What advice do you have for other primary care clinicians – How can they better serve axial spondyloarthritis patients? |
| **Implementing a screening tool** | If there were a validated screening tool for axial spondyloarthritis – what would help you to use it?  - what would deter you from using it? |
