# Supplementary material for: Primary care physician perspectives on screening for axial spondyloarthritis: A qualitative study
Source: PLoS One. 2021 May 24;16(5):e0252018. doi: 10.1371/journal.pone.0252018 (PMC8143395; doi:10.1371/journal.pone.0252018)
Supplement: Attachment — Submitted filename: Plos.docx [file pone.0252018.s003.docx]

none
