# Supplementary material for: Primary care physician perspectives on screening for axial spondyloarthritis: A qualitative study
Source: PLoS One. 2021 May 24;16(5):e0252018. doi: 10.1371/journal.pone.0252018 (PMC8143395; doi:10.1371/journal.pone.0252018)
Supplement: Attachment — Submitted filename: PONE-D-20-26736_Response to Reviewers.docx [file pone.0252018.s004.docx]

**Journal Requirements:**

**When submitting your revision, we need you to address these additional requirements.**

**1. Please ensure that your manuscript meets PLOS ONE's style requirements, including those for file naming. The PLOS ONE style templates can be found at**

**https://journals.plos.org/plosone/s/file?id=wjVg/PLOSOne_formatting_sample_main_body.pdf and**

**https://journals.plos.org/plosone/s/file?id=ba62/PLOSOne_formatting_sample_title_authors_affiliations.pdf**

We have reviewed the style requirements and revised accordingly.

**2. Please provide additional details regarding participant consent. In the ethics statement in the Methods and online submission information, please ensure that you have specified (1) whether consent was informed and (2) what type you obtained (for instance, written or verbal, and if verbal, how it was documented and witnessed). If the need for consent was waived by the ethics committee, please include this information.**

**Once you have amended this/these statement(s) in the Methods section of the manuscript, please add the same text to the “Ethics Statement” field of the submission form (via “Edit Submission”).**

**For additional information about PLOS ONE ethical requirements for human subjects research, please refer to http://journals.plos.org/plosone/s/submission-guidelines#loc-human-subjects-research.**

We have revised and provided further details regarding the consent process in the Methods section.

**3. In your Methods section, please provide additional information about the participant recruitment method and the demographic details of your participants. Please ensure you have provided sufficient details to replicate the analyses such as: a) the recruitment date range (month and year), and b) a description of any inclusion/exclusion criteria that were applied to participant recruitment.**

We have revised provided additional details on the recruitment process.

**4. Thank you for stating the following in the Financial Disclosure section:**

**"Funding for this project was provided by Novartis Pharmaceuticals Corporation. Research reported in this publication was supported by the National Center for Advancing Translational Sciences of the National Institutes of Health under award number UL1TR000161 (KLL). This work was also supported by a charitable contribution to the UMass Memorial Foundation from Timothy S. and Elaine L. Peterson (JK, SHL). This project was also supported by the SAA/Jane Bruckel Early Career Investigator in AxSpA Award (SHL). The content is solely the responsibility of the authors and does not necessarily represent the official views of the NIH.** **The funders had no role in study design, data collection and analysis, decision to publish, or preparation of the manuscript."**

**We note that one or more of the authors have an affiliation to the commercial funders of this research study : Novartis Pharmaceuticals Corporation.**

**4.1. Please provide an amended Funding Statement declaring this commercial affiliation, as well as a statement regarding the Role of Funders in your study. If the funding organization did not play a role in the study design, data collection and analysis, decision to publish, or preparation of the manuscript and only provided financial support in the form of authors' salaries and/or research materials, please review your statements relating to the author contributions, and ensure you have specifically and accurately indicated the role(s) that these authors had in your study. You can update author roles in the Author Contributions section of the online submission form.**

**Please also include the following statement within your amended Funding Statement.**

**“The funder provided support in the form of salaries for authors [insert relevant initials], but did not have any additional role in the study design, data collection and analysis, decision to publish, or preparation of the manuscript. The specific roles of these authors are articulated in the ‘author contributions’ section.”**

**If your commercial affiliation did play a role in your study, please state and explain this role within your updated Funding Statement.**

**4.2. Please also provide an updated Competing Interests Statement declaring this commercial affiliation along with any other relevant declarations relating to employment, consultancy, patents, products in development, or marketed products, etc.**

**Within your Competing Interests Statement, please confirm that this commercial affiliation does not alter your adherence to all PLOS ONE policies on sharing data and materials by including the following statement: "This does not alter our adherence to PLOS ONE policies on sharing data and materials.” (as detailed online in our guide for authors http://journals.plos.org/plosone/s/competing-interests). If this adherence statement is not accurate and there are restrictions on sharing of data and/or materials, please state these. Please note that we cannot proceed with consideration of your article until this information has been declared.**

**Please include both an updated Funding Statement and Competing Interests Statement in your cover letter. We will change the online submission form on your behalf.**

**Please know it is PLOS ONE policy for corresponding authors to declare, on behalf of all authors, all potential competing interests for the purposes of transparency. PLOS defines a competing interest as anything that interferes with, or could reasonably be perceived as interfering with, the full and objective presentation, peer review, editorial decision-making, or publication of research or non-research articles submitted to one of the journals. Competing interests can be financial or non-financial, professional, or personal. Competing interests can arise in relationship to an organization or another person. Please follow this link to our website for more details on competing interests:** [**http://journals.plos.org/plosone/s/competing-interests**](http://journals.plos.org/plosone/s/competing-interests)

We have updated the statements in the online submission form and cover letter.

**5. We note that you have indicated that data from this study are available upon request. PLOS only allows data to be available upon request if there are legal or ethical restrictions on sharing data publicly. For information on unacceptable data access restrictions, please see http://journals.plos.org/plosone/s/data-availability#loc-unacceptable-data-access-restrictions.**

**In your revised cover letter, please address the following prompts:**

**a) If there are ethical or legal restrictions on sharing a de-identified data set, please explain them in detail (e.g., data contain potentially identifying or sensitive patient information) and who has imposed them (e.g., an ethics committee). Please also provide contact information for a data access committee, ethics committee, or other institutional body to which data requests may be sent.**

**b) If there are no restrictions, please upload the minimal anonymized data set necessary to replicate your study findings as either Supporting Information files or to a stable, public repository and provide us with the relevant URLs, DOIs, or accession numbers. Please see http://www.bmj.com/content/340/bmj.c181.long for guidelines on how to de-identify and prepare clinical data for publication. For a list of acceptable repositories, please see http://journals.plos.org/plosone/s/data-availability#loc-recommended-repositories.**

**We will update your Data Availability statement on your behalf to reflect the information you provide.**

We have revised and provided a statement regarding data availability in both cover letter and manuscript.

**6. Please include captions for your Supporting Information files at the end of your manuscript, and update any in-text citations to match accordingly. Please see our Supporting Information guidelines for more information:** [**http://journals.plos.org/plosone/s/supporting-information**](http://journals.plos.org/plosone/s/supporting-information)**.**

We have revised and include captions for our supporting information.

**Review Comments to the Author**

**Reviewer #1:**

**I thank the authors the work they did and method they used to respond to their research question. However, their work need to be reviewed before it is published. Below are some observations and comments:**

We thank the reviewer for their thoughtful review and helpful comments. We believe the manuscript is stronger with the suggested revisions. Please note that these paragraph and page numbers correspond with the version of the revised manuscript with tracked changes.

**1. Line 60-61: In the background you mention that the diagnostic delay is 5 to 14 years in while in the introduction of the manuscript you mention 5 years or longer. I expected to see the same in the manuscript, which I did not. Moreover, cross check your reference. It is not clear to me how a published manuscript on the same study with the same authors published very recently mentions 5 to 10, and in this study, you mention 5 to 14. Just to double check if it is in line with the used references or there may be any inaccuracy.**

Author response: We regret the confusion.

Author action: We have revised and updated the references accordingly.

**2. Line 69: “the modified New York” sounds incomplete; add the word that may be missing; criteria or tool??**

Author response: We thank the reviewer for the careful review of the manuscript.

Author action: We have revised.

**3. Why did you purposively recruited primary health care physicians and internal medicine specialists? What was behind that purpose? Can you add something on setting sections about how the system works? Just for a reader who is unfamiliar with US health system.**

Author response: We regret the confusion. The reasons contributing to diagnostic delay in patients with axSpA is multi-factorial. First, the primary/initial symptom of axSpA is low back pain. When axSpA patients initially have their symptoms (i.e., back pain or spinal pain), they often consult with primary care providers (e.g., primary care physicians or internalist) seeking symptom relief. However, low back pain is common in the US primary care settings. The commonly seen mechanical back pain in the US has led to an uncommon diagnosis such axSpA being missed or considered late in the disease and thus resulting in the diagnostic delay. Second, awareness of SpA is low among US non-rheumatologist physicians. With regards the clinical practice, only 26% of patients with ankylosing spondylitis in the US are diagnosed by primary care physicians despite that patients often first seek care in the primary care settings (Deodhar A, et. al. 2016). Our study aimed to address this critical research gap where we solicited feedback from primary care physicians including PCPs and internalist regarding questions from the Inflammatory Back Pain Assessment: the Assessment of Spondyloarthritis International Society (ASAS) Expert Criteria and gained insight about barriers and facilitators for potentially implementing a screening tool for patients with axSpA in primary care settings.

Author action: We have revised the Introduction and the sentence for clarity to read: “Although rheumatologists play a pivotal role in caring for patients with axSpA, most patients often consult with primary care providers (e.g., primary care physicians or internalist) seeking symptom relief when they initially have their symptoms (i.e., back pain or spinal pain) and are diagnosed by primary care providers other than rheumatologists. [Page 3, line 72-77]”

**4. Minimize the use of the passive voice wherever it is possible.**

Author response: We thank the reviewer for the comment. We have carefully reviewed and edited the manuscript throughout.

**5. Why didn’t you keep this question qualitative? “How confident are you in distinguishing inflammatory back pain from mechanical back pain?”**

Author response: We regret the confusion. This question was part of the structured questionnaires which was administered at the end of the in-depth interview. The questionnaire includes items about socio-demographics, practice characteristics, awareness of the features of inflammatory back pain according to several different sets of classification criteria. The question was used to understand the characteristics of physician participants for the study.

Author action: We have revised to address the reviewer’s comment.

**6. Table 5 and 6 show who said what, that is not done for tables 3, and 4. It would be better if you keep it consistent. It also looks like the participants’ language on main themes 1 and 2 is missing. In qualitative studies, I expect to read partipants’ own words instead of rephrased sentences. Keep the same style for all themes.**

Author response: We regret the confusion.

Author action: We have revised accordingly and presented the representative quotes from the participants for all themes, including tables 3 and 4.

**7. I appreciate the introductions to each subtheme. However, it would be better if you do not separate them from the exerpts you put in the tables. Using a narrative style as if you are telling your story makes it easier for the reader. You do not have to change if you prefer that structure.**

Author response: We thank the reviewer for the comment. We consider it may be difficult for the reader to process how the results all fit together in the manuscript by using the narrative style to present the findings. As such, we present the synthesis of the findings by introducing the themes and subthemes of our findings.

**8. Please elaborate more on the first theme in both result and discussion sections.**

Author response: We agree.

Author action: We have revised and expanded the contents in both sections. We have also added represented quotes in Table 3.

We have revised the sentences below for clarity to read:

“When asking of the typical baseline work-up of axSpA patients (Table 3), physicians noted several clinical observations potentially pointing to axSpA, including: 1) young people presenting with back symptoms without any antecedent injury; 2) the presence of comorbid autoimmune conditions; 3) peripheral joint involvement, and other systemic manifestations (e.g., iritis). When speaking of the role of clinical examinations and tests, most physicians stressed that a thorough medical history would be essential for diagnosing axSpA.” [Page 8, 161-166]

“Physicians are concerned about overuse of testing and low-value healthcare given that dissatisfied patients when costly tests are performed that may not yield definitive results.[29] Primary care physicians have difficulty discriminating inflammatory back pain from other kinds of back pain and are often unaware of other SpA features that are important for the differential diagnosis. Add to this fact that the radiological proof is sometimes a late feature of the disease and the result is the diagnostic delay.” [Page 15, line 214-218]

**Reviewer #2:**

**This is a research to answer a relevant question to primary healthcare providers. The choice of the study design was good and the analysis of the data is adequate.**

**Some bias exist inevitably, but this was highlighted well in the discussion section. I do not think that this affect the quality of the research or the validity of the results.**

We thank the reviewer for noting the relevance of the research questions and adequate methods used. We believe the paper has been improved and strengthened during its revision.

**Reviewer #3:**

**The am this study to solicit feedback from primary care physicians regarding questions from the Inflammatory Back Pain Assessment: the Assessment of Spondyloarthritis International Society (ASAS) Expert Criteria and gain insight about barriers and facilitators for implementing axSpA screening in primary care. This is a very important study.**

We appreciate the kind and positive feedback about our manuscript from the reviewer.

**Reviewer #4:**

**There article is nicely written.**

**The authors have presented a qualitative research (study instrument being in-depth interviews with 18 primary care physicians) to explore primary care physicians' perspective on screening for axial spondyloarthritis.**

We thank the reviewer for the evaluation of our work. We have made revisions according to the comments and suggestions.

**There are following observations/concerns:**

**1. Most of the physicians recruited for the study were known to the authors. Considering nature of the study, this could have introduced interpretation bias among the recruited physician(s).**

Author response: We agree and thank the reviewer for the feedback. Indeed, a convenience sample was used for the present study. However, despite that some of the physicians were known to the authors, the interviewer (KLL) was not someone with a prior relationship with the participants and therefore the bias could be reduced.

Author action: We have addressed this concern and expanded the section of strengths and limitations.

**2. None of the physicians recalled ever having diagnosed a patient with axial spondyloarthritis.**

Author response: We agree thank the reviewer for the comment. From out background questionnaire, we found that most participants were confident in distinguishing inflammatory versus mechanical back pain. Further, the average years in practice was 15.7 years and the majority were affiliated with academic institutions.

Author action: We have revised and expanded the section of strengths and limitations.
